# Supplementary material for: Temporal Gene Expression Profiles Reflect the Dynamics of Lymphoid Differentiation
Source: Int J Mol Sci. 2022 Jan 20;23(3):1115. doi: 10.3390/ijms23031115 (PMC8834919; doi:10.3390/ijms23031115)
Supplement: Supplementary file 1 [file ijms-23-01115-s001.zip › Data Sheet 2.pdf]

Total RNA-seq data processing

Total RNA stranded sequenced reads were trimmed using seqtk trimfq (https://github.com/lh3/seqtk, version 20141003, with parameter b:4) to remove the first three nucleotides which corresponded to sequencing adapter and the fourth one which was of low quality. Then, 3'-end sequencing adapters and low quality bases were trimmed using Trimmomatic (version 0.32, with parameters illuminaClip:2:30:10, leading:30, trailing:30, slidingWindow:4:30, minlen:30) (Bolger et al., 2014). Trimmed reads were then aligned on mouse reference genome (GRCM38 ENSEMBL release 75) using Tophat2 (version 2.0.13, with parameters min-anchor-length:6, splice-mismatches:1, b2-very-sensitive, mate-std-dev:100, read-mismatches:4, read-gap-length:3, read-edit-dist:5) (Kim et al., 2013). Quality controls were performed from a sampling of 2x10 millions reads per replicate using Picard suite (version 1.122, multipleMetrics, http://broadinstitute.github.io/picard/) and RNA-SeQC (DeLuca et al., 2012). Read counts per gene were performed using HTSeq-count (version 0.6.1, with parameters stranded:yes, type:exon, idattr:gene_id, mode:union) (Anders et al., 2015). This bioinformatic pipeline was implemented using BPIPE workflow manager (version 0.9.8.6) (Sadedin et al., 2012).

Gene expression analysis

Read counts per gene were normalized by log2(FPKM+1) (Fragments Per gene Kilobase and per Million reads) and adjusted using comBat from Bioconductor sva package to remove technical variability between batches of mice (Johnson et al., 2007; Leek et al., 2019). These normalized and adjusted gene quantification data were assessed by hierarchical clustering (hclust R package) to test for reproducibility between biological replicates of the same cell type (Müllner, 2013). Distribution of gene biotype quantification was carried out using Bioconductor rtracklayer package (Lawrence et al., 2009).

Differential expression analyses between pairs of progenitor cell populations were performed, from raw read counts per gene, using DESeq2 with batch effects included in design formula (Love et al., 2014).

Genes were considered as significantly differentially expressed if their corrected P-value was lower than 5% and their absolute log2 fold change greater than 0.5 (Schurch et al., 2016).

Gene ontology was used to select, among differentially expressed genes, those corresponding to cell surface marker or transcription factor. Genes were annotated as cell surface protein if their cellular component contained terms of 'membrane' or 'cell surface' and if their molecular function contained terms of 'receptor' or 'binding'. Genes were annotated as transcription factor if their molecular function contained term of 'transcription factor activity'. This ontological annotation was carried out using biomaRt library from Bioconductor (version feb2014) (Durinck et al., 2005).

Unsupervised gene classification was performed from differentially expressed genes to identify groups of genes co-expressed during differentiation. The similarity matrix, based on Person correlation coefficient, was calculated using Dist function from Amap R package (`https://CRAN.R-project.org/package=amap`). Hierarchical clustering was generated using hclust function from Hmisc R package, by maximizing the average linkage distance between clusters (`https://CRAN.R-project.org/package=Hmisc`). A dendrogram cut into two classes was used to discriminate genes with increased or decreased expression profile during hematopoietic differentiation.

Identification of over-represented biological pathways in genes of interest was performed using over-representation test (Released 2019-07-11) of the PantherDB (version 14.1 Released 2019-03-12) (Mi et al., 2017). List of differentially expressed genes (DEG) identified from comparison between pairs of progenitor populations and genes with increase or decrease expression profile across differentiation were alternatively considered for these pathway over-representation analysis. Reactome (version 65 Released 2019-03-12) and PantherDB (Pathway 3.6.3 Released 2019-03-12) have been used as pathway annotation resources (Fabregat et al., 2016). All over-representation analyses were performed relative to the 22,296 Mus musculus genes (MGI released 2018-04). Pathways were considered as significantly

over-/under-represented if they were associated with a corrected P-value lower than 5%, after Fisher's Exact test with FDR multiple test correction.

Gene regulation analysis

In silico sequence prediction of transcription factor binding sites (TFBS) was performed on the 1kbp genomic region upstream to transcription start site from each mouse gene. Gene promoter sequences were retrieved using biomaRt library from Bioconductor (version feb2014) (Durinck et al., 2005). Motif enrichment analysis was carried out using AME method (Analysis of Motif Enrichment, Version 4.11.3) from the MEME tool suite (McLeay and Bailey, 2010) and the collection of TFBS position frequency matrices referenced in HOCOMOCO Mouse database (v10 release) (Kulakovskiy et al., 2016). Comparison between a gene group of interest and its shuffled version allowed us to identify enriched TFBSs. A motif was considered as significantly enriched in a gene group if its P-value was lower than 5%, after Bonferroni correction for multiple testing. Then to identify genes regulated by an enriched TFBS, motif scanning was carried out on promoter sequences using MAST method (Motif Alignment & Search Tool, Version 4.11.3) (Bailey et al., 2015) and HOCOMOCO Mouse (v10 release) (Kulakovskiy et al., 2016). We conserved TFBS predicted by MAST if they were associated with an E-value lower than 250.

Gene regulatory networks connecting surface markers (SMs) and transcription factors (TFs) were modeled using Cytoscape software (version 3.7.0) (Shannon et al., 2003). The genes are represented by nodes and predicted regulatory interaction between genes by edges. The node shapes represent TFs (with diamonds or triangles) or SMs (with hexagons). The node border colors annotate genes that are up-regulated (in red) or down-regulated (in blue) during lymphoid differentiation. The node background colors illustrate the genes known to be involved in B cell lymphopoiesis (in orange), in T cell lymphopoiesis (in purple) or in myelopoiesis (in green). The nodes were connected by edges according

to our predicted regulatory relationships between them using edges starting from a TF and pointing to a SM or another TF. An arbitrarily color was assigned to outgoing edges connected to the same TF in order to facilitate network visualization.

Prediction of novel long non-coding RNAs (lncRNA)

RNA-seq read alignments produced by Tophat2 from each biological replicate and from each cell type (MPP2, MPP3, CLP) were concatenated into a single alignment file to search for novel long non-coding RNAs. Genome-guided transcript assembly was performed, on this concatenated BAM file, by the Cufflinks suite (2.2.2.20150820git) (Trapnell et al., 2012). Transcript assemblies which did not correspond to an annotated Mouse gene (GRCM38 ENSEMBL release 75) were considered as novel. Moreover, only predicted transcripts longer than 200bp and made of less than 5 exons were conserved. Genomic coordinates of these novel transcripts were compared against Mouse gene annotation (GRCM38 ENSEMBL release 75) using Bedtools suite to identify novel loci located in intronic regions of annotated genes or in inter-genic region and to retrieve the corresponding fasta sequences (Quinlan and Hall, 2010). Potential novel transcripts overlapping the genomic coordinates and sharing the same strand as annotated transcripts were not kept. The CPAT tool (Coding-Potential Assessment Tool) was used to assess the coding potential of each novel transcript (logit model and hexamer frequency table files provided with CPAT) (Wang et al., 2013). A transcript assigned to a CPAT coding probability threshold lower than 0.44, the default value for mouse, and with a minimum length of 200bp was considered as potential novel long-non coding RNA. Then, genomic coordinates of these novel transcripts, validated by CPAT, were concatenated with mouse reference annotated genes to generate a common GTF file describing all annotated genes and putative long non-coding RNAs. This GTF was used with HTSeq-count and read alignments to generate a read count per gene separately for each biological replicate of each cell type (Anders et al., 2015). Differential gene expression analyses were

finally performed between pairs of progenitor cell populations using DESeq2, as previously described for protein coding genes, in order to identify novel long-non coding RNAs associated to specific cell type (Love et al., 2014).

References

Anders, S., Pyl, P.T., and Huber, W. (2015). HTSeq--a Python framework to work with high-throughput sequencing data. Bioinforma. Oxf. Engl. *31*, 166–169.

Bailey, T.L., Johnson, J., Grant, C.E., and Noble, W.S. (2015). The MEME Suite. Nucleic Acids Res. *43*, W39–W49.

Bolger, A.M., Lohse, M., and Usadel, B. (2014). Trimmomatic: a flexible trimmer for Illumina sequence data. Bioinforma. Oxf. Engl. *30*, 2114–2120.

DeLuca, D.S., Levin, J.Z., Sivachenko, A., Fennell, T., Nazaire, M.-D., Williams, C., Reich, M., Winckler, W., and Getz, G. (2012). RNA-SeQC: RNA-seq metrics for quality control and process optimization. Bioinforma. Oxf. Engl. *28*, 1530–1532.

Durinck, S., Moreau, Y., Kasprzyk, A., Davis, S., De Moor, B., Brazma, A., and Huber, W. (2005). BioMart and Bioconductor: a powerful link between biological databases and microarray data analysis. Bioinforma. Oxf. Engl. *21*, 3439–3440.

Fabregat, A., Sidiropoulos, K., Garapati, P., Gillespie, M., Hausmann, K., Haw, R., Jassal, B., Jupe, S., Korninger, F., McKay, S., et al. (2016). The Reactome pathway Knowledgebase. Nucleic Acids Res. *44*, D481-487.

Johnson, W.E., Li, C., and Rabinovic, A. (2007). Adjusting batch effects in microarray expression data using empirical Bayes methods. Biostat. Oxf. Engl. *8*, 118–127.

Kim, D., Pertea, G., Trapnell, C., Pimentel, H., Kelley, R., and Salzberg, S.L. (2013). TopHat2: accurate alignment of transcriptomes in the presence of insertions, deletions and gene fusions. Genome Biol. *14*, R36.

Kulakovskiy, I.V., Vorontsov, I.E., Yevshin, I.S., Soboleva, A.V., Kasianov, A.S., Ashoor, H., Ba-alawi, W., Bajic, V.B., Medvedeva, Y.A., Kolpakov, F.A., et al. (2016). HOCOMOCO: expansion and enhancement of the collection of transcription factor binding sites models. Nucleic Acids Res. *44*, D116–D125.

Lawrence, M., Gentleman, R., and Carey, V. (2009). rtracklayer: an R package for interfacing with genome browsers. Bioinforma. Oxf. Engl. *25*, 1841–1842.

Leek, J., Johnson, W., Parker, H., Fertig, E., Jaffe, A., Storey, J., Zhang, Y., and Torres, L. (2019). sva: Surrogate Variable Analysis. R Package Version 3301.

Love, M.I., Huber, W., and Anders, S. (2014). Moderated estimation of fold change and dispersion for RNA-seq data with DESeq2. Genome Biol. *15*.

McLeay, R.C., and Bailey, T.L. (2010). Motif Enrichment Analysis: a unified framework and an evaluation on ChIP data. BMC Bioinformatics *11*, 165.

Mi, H., Huang, X., Muruganujan, A., Tang, H., Mills, C., Kang, D., and Thomas, P.D. (2017). PANTHER version 11: expanded annotation data from Gene Ontology and Reactome pathways, and data analysis tool enhancements. Nucleic Acids Res. *45*, D183–D189.

Müllner, D. (2013). fastcluster: Fast Hierarchical, Agglomerative Clustering Routines for R and Python. J. Stat. Softw. *53*, 1–18.

Quinlan, A.R., and Hall, I.M. (2010). BEDTools: a flexible suite of utilities for comparing genomic features. Bioinforma. Oxf. Engl. *26*, 841–842.

Sadedin, S.P., Pope, B., and Oshlack, A. (2012). Bpipe: a tool for running and managing bioinformatics pipelines. Bioinforma. Oxf. Engl. *28*, 1525–1526.

Schurch, N.J., Schofield, P., Gierliński, M., Cole, C., Sherstnev, A., Singh, V., Wrobel, N., Gharbi, K., Simpson, G.G., Owen-Hughes, T., et al. (2016). How many biological replicates are needed in an RNA-seq experiment and which differential expression tool should you use? RNA *22*, 839–851.

Shannon, P., Markiel, A., Ozier, O., Baliga, N.S., Wang, J.T., Ramage, D., Amin, N., Schwikowski, B., and Ideker, T. (2003). Cytoscape: A Software Environment for Integrated Models of Biomolecular Interaction Networks. Genome Res. *13*, 2498–2504.

Trapnell, C., Roberts, A., Goff, L., Pertea, G., Kim, D., Kelley, D.R., Pimentel, H., Salzberg, S.L., Rinn, J.L., and Pachter, L. (2012). Differential gene and transcript expression analysis of RNA-seq experiments with TopHat and Cufflinks. Nat. Protoc. *7*, 562–578.

Wang, L., Park, H.J., Dasari, S., Wang, S., Kocher, J.-P., and Li, W. (2013). CPAT: Coding-Potential Assessment Tool using an alignment-free logistic regression model. Nucleic Acids Res. *41*, e74–e74.
