# Supplementary material for: Temporal Gene Expression Profiles Reflect the Dynamics of Lymphoid Differentiation
Source: Int J Mol Sci. 2022 Jan 20;23(3):1115. doi: 10.3390/ijms23031115 (PMC8834919; doi:10.3390/ijms23031115)
Supplement: Supplementary file 1 [file ijms-23-01115-s001.zip › Data Sheet 3.pdf]

Figure S1. Bioinformatic methodology used to analyze RNAseq profiling of lymphoid progenitors.

The workflow used to analyse the transcriptomes of lymphoid progenitors is made of four parts : (i) the **quality control panel** describes the quality assessment of RNAseq reads mapped to the mouse reference genome; (ii) the **gene quantification panel** describes the pre-processing of reads, their genome mapping, the estimation of gene abundance and the correction of batch effect; (iii) the **differential expression and classification panel** details the identification of differentially expressed genes (DEGs), their classification and the detection of biological pathways over-represented in DEGs; and (iv) the **gene regulation panel** explains the steps used to predict a core gene regulatory network associated with the lymphoid differentiation.

Figure S2. Unsupervised clustering of progenitor cells from gene expression.

Unsupervised hierarchical clustering of progenitor populations from expressed genes was carried out before (a) and after (b) batch effect correction with ComBat method. (a) Clustering analysis from gene expression values with FPKM greater than 0.1 in at least one biological replicate and transformed with log2(FPKM+1). (b) Clustering analysis from gene expression values with FPKM greater than 0.1 in at least one biological replicate, transformed with log2(FPKM+1) and corrected for batch effect using ComBat method.

Figure S3. Classification of differentially expressed surface markers and transcription factors.

Heatmap showing the abundance values of the surface markers and transcription factors differentially expressed in a least one pairwise comparison of progenitor populations. Unsupervised hierarchical clustering was used to classify these genes in two clusters according to their expression pattern during progenitor differentiation. Up-regulated genes are grouped in cluster 1 whereas down-regulated genes

are grouped in cluster 2. Surface antigens already known to be involved in lymphoid differentiation were annotated.

Figure S4. Validation by RT-PCR of protein coding genes expressed during lymphoid differentiation.

RT-PCR results showing the presence or absence expression of 6 selected genes. Eight experiments (wells) were performed per progenitor population.

Figure S5. Exploration of transcription factors.

Heatmap showing the abundance values of the transcription factors differentially expressed between MPP2 and CLP cells. The bar plot (right panel) illustrates the Log2 fold change per gene.

Figure S6. Biological pathways associated with lymphoid commitment.

Bar plots illustrating pathway enrichment analysis from surface markers and transcription factors up-regulated (red) or down-regulated (blue) between MPP2 and CLP cells.

Figure S7. Validation by RT-PCR of novel long non-coding RNAs expressed during lymphoid differentiation.

RT-PCR results showing expression status of six novel lncRNAs. Eight experiments (wells) were performed per progenitor population.
