# Supplementary figures and images for: Temporal Gene Expression Profiles Reflect the Dynamics of Lymphoid Differentiation
Source: Int J Mol Sci. 2022 Jan 20;23(3):1115. doi: 10.3390/ijms23031115 (PMC8834919; doi:10.3390/ijms23031115)

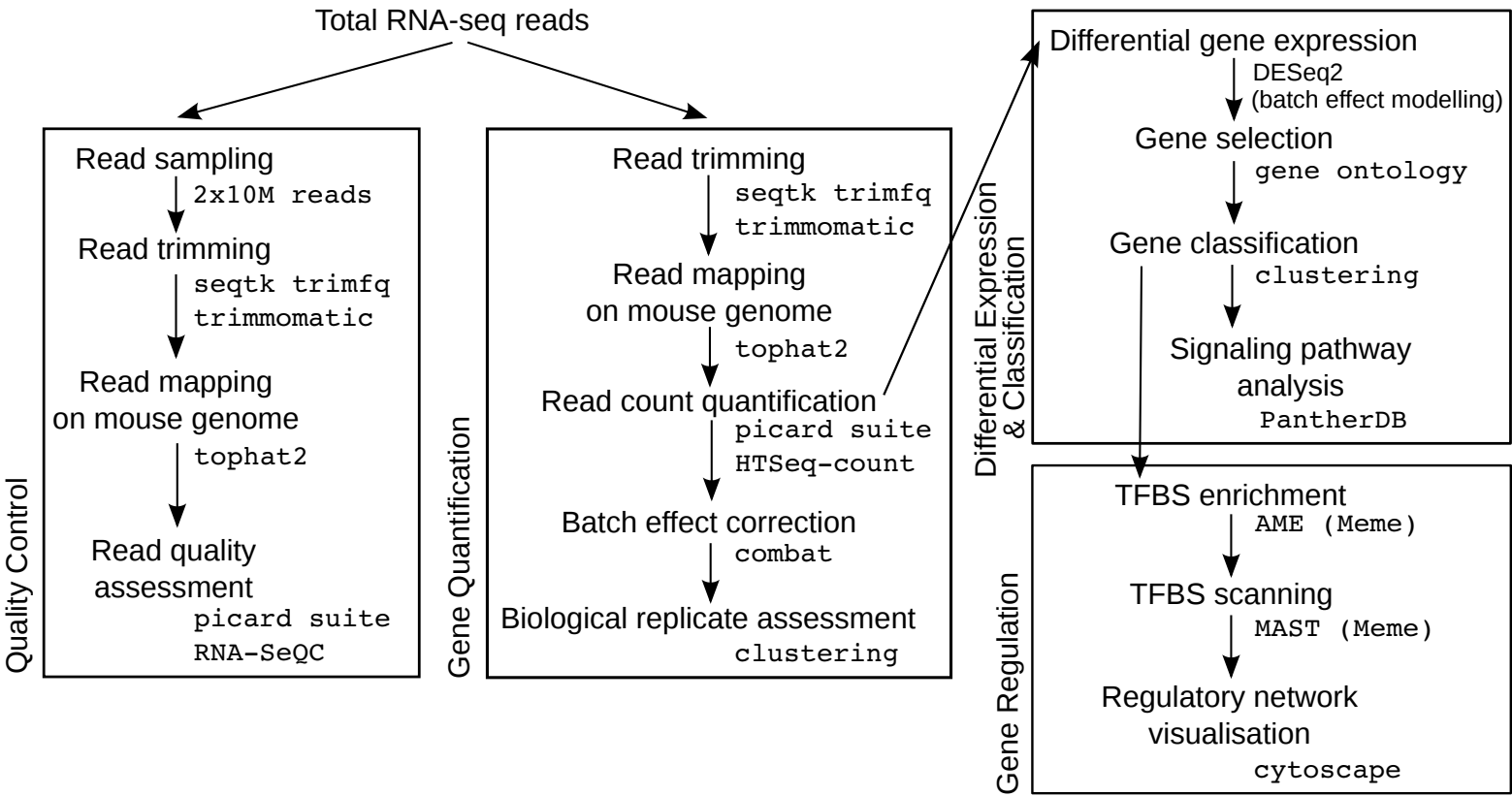

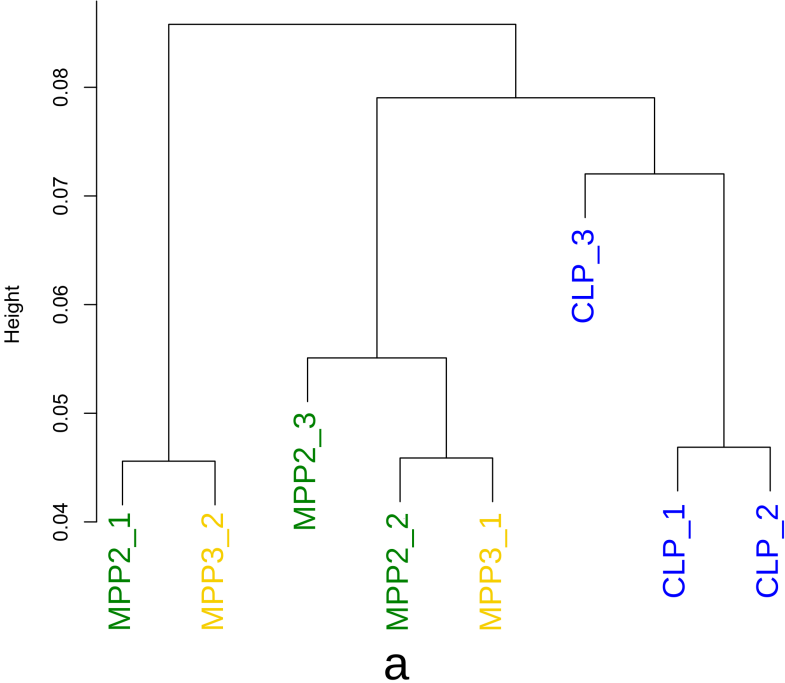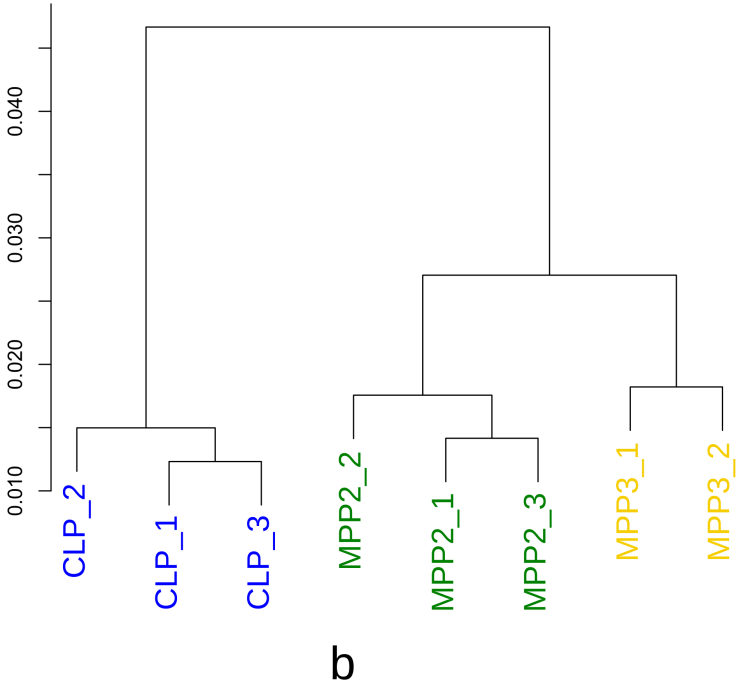

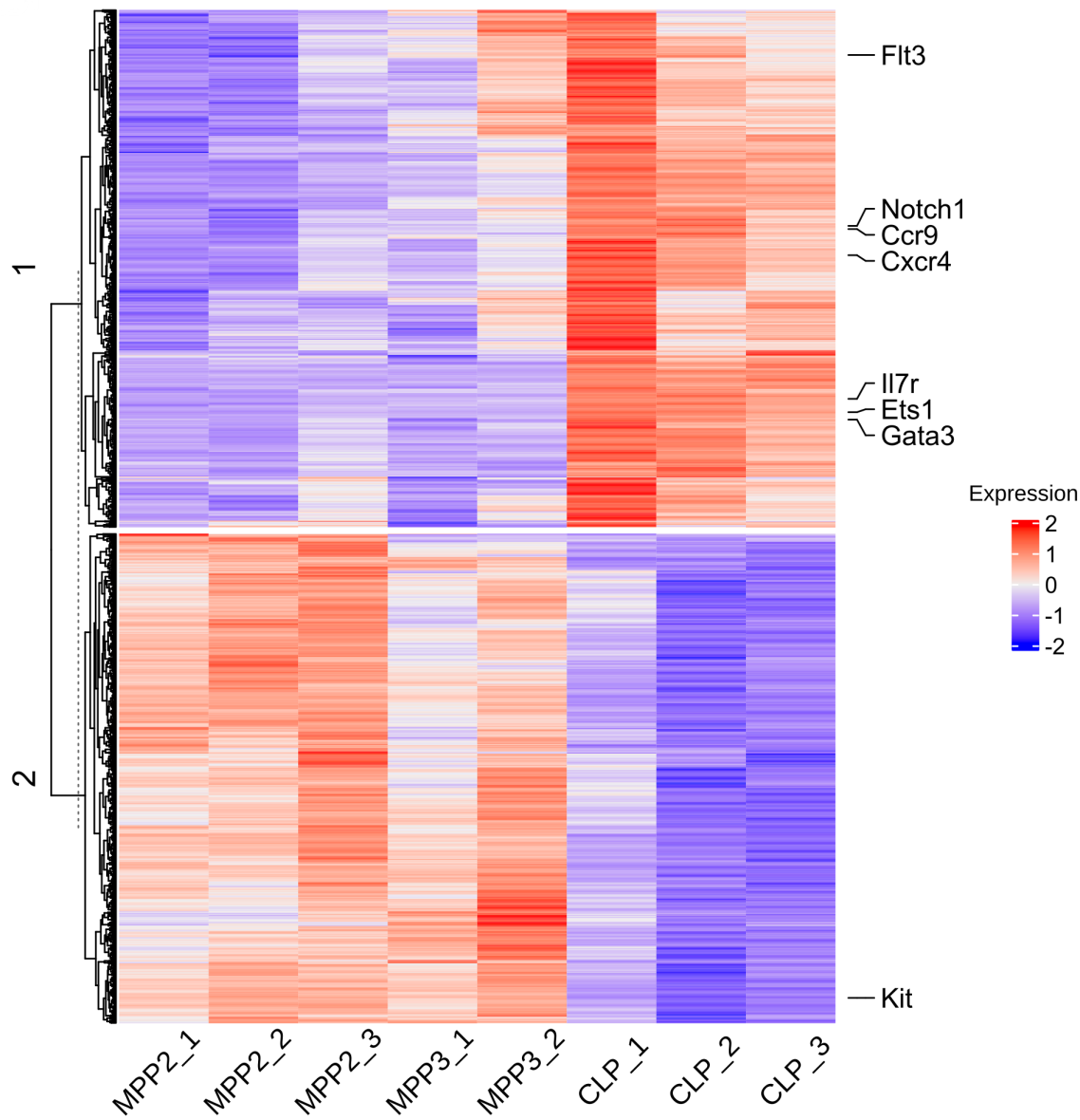

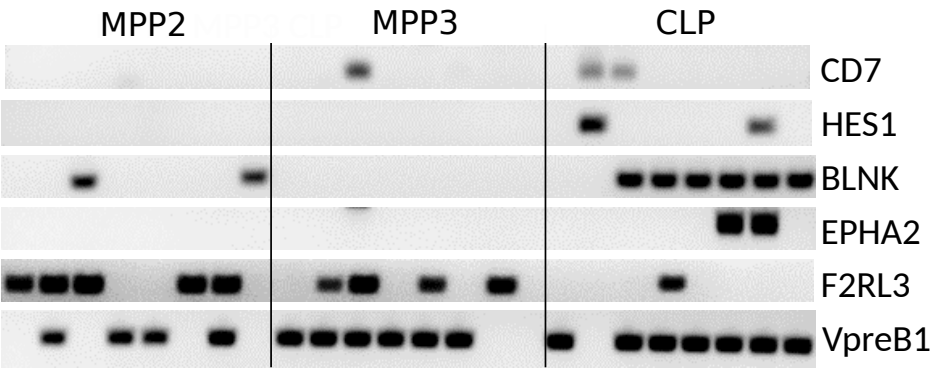

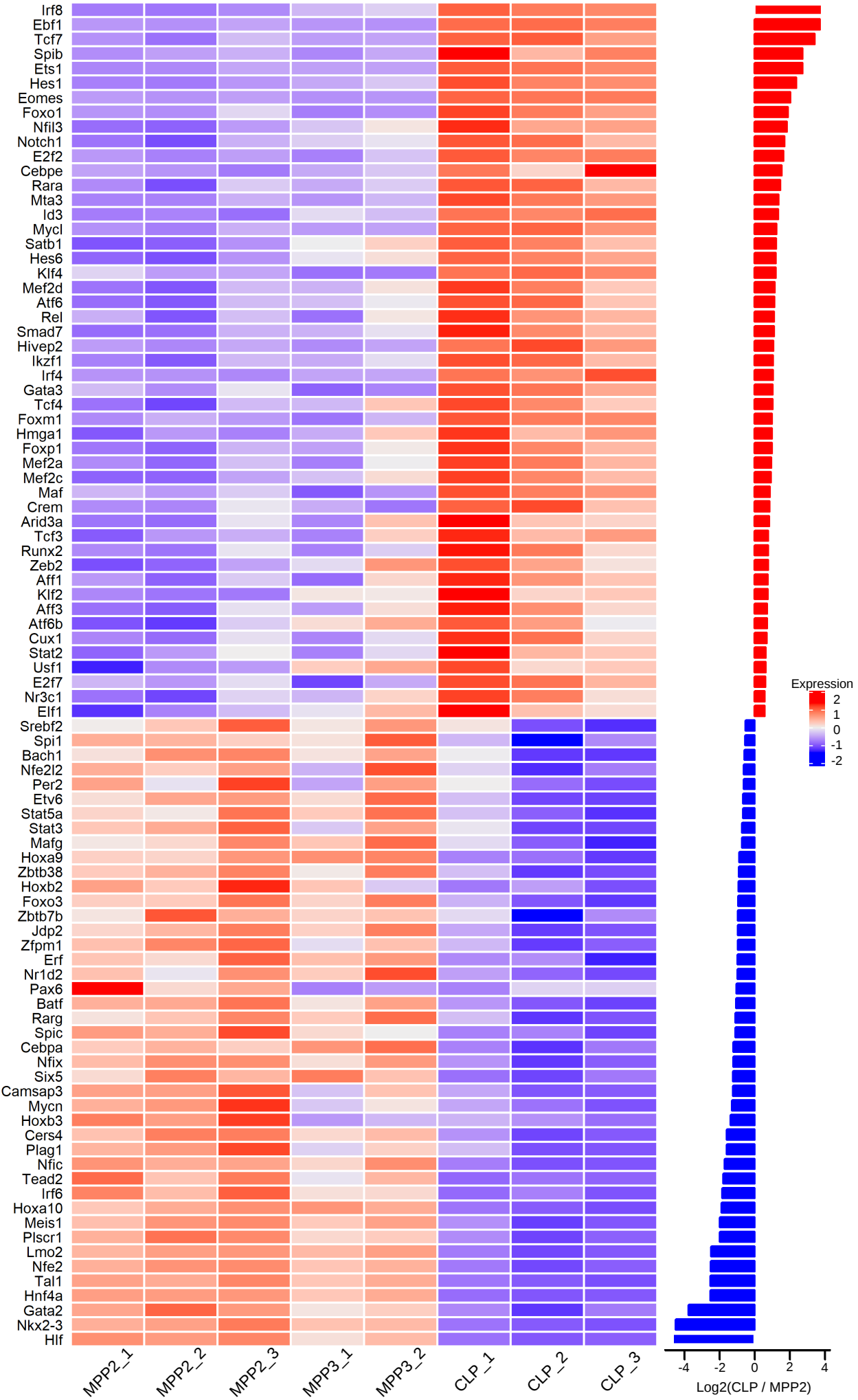

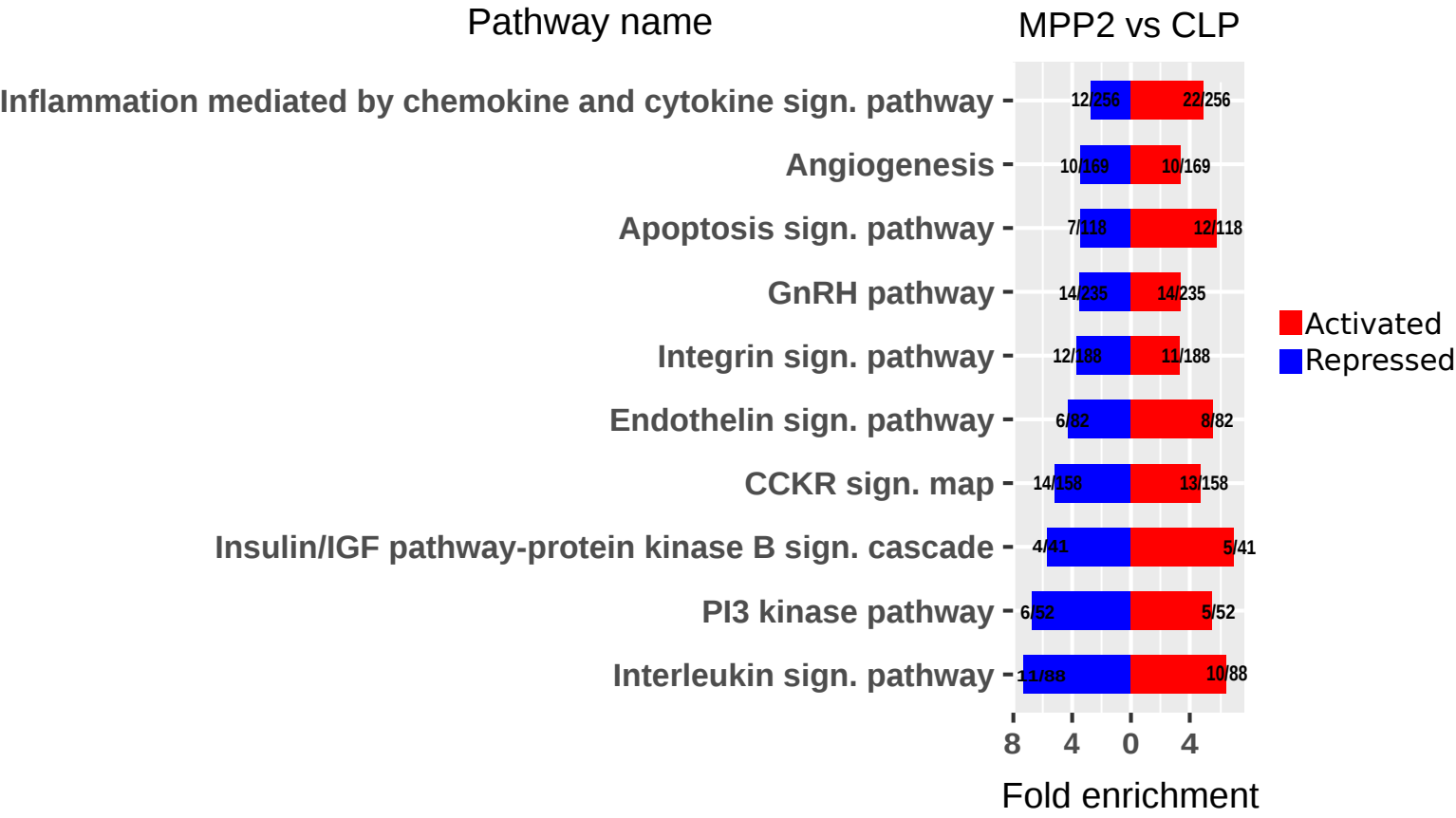

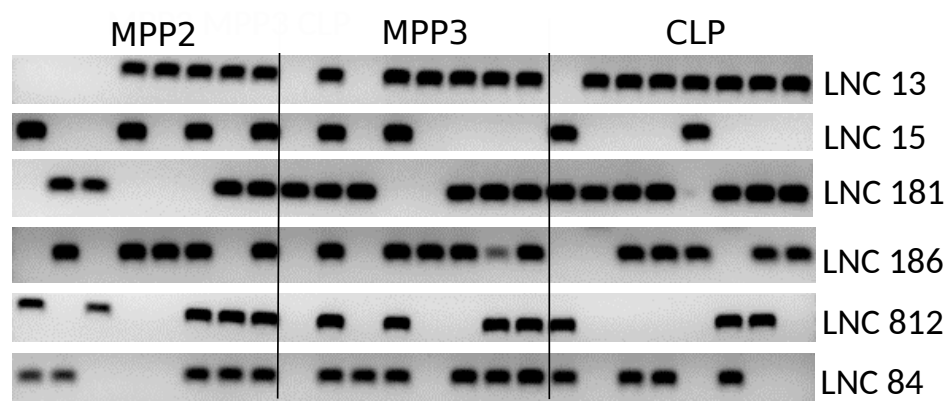

Supplement: Supplementary file 1 [file ijms-23-01115-s001.zip › Data Sheet 1.pdf]
